# Electrically induced transformations of defects in cholesteric layer with tangential-conical boundary conditions

**DOI:** 10.1038/s41598-020-61713-9

**Published:** 2020-03-17

**Authors:** Mikhail N. Krakhalev, Oxana O. Prishchepa, Vitaly S. Sutormin, Rashid G. Bikbaev, Ivan V. Timofeev, Victor Ya. Zyryanov

**Affiliations:** 1 Kirensky Institute of Physics, Federal Research Center KSC SB RAS, Krasnoyarsk, 660036 Russia; 2 Institute of Engineering Physics and Radio Electronics, Siberian Federal University, Krasnoyarsk, 660041 Russia

**Keywords:** Liquid crystals, Liquid crystals

## Abstract

Electric-field-induced changes of the orientational structures of cholesteric liquid crystal layer with the tangential-conical boundary conditions have been investigated. The samples with the ratio of the cholesteric layer thickness *d* to the helix pitch *p* equalled to 0.57 have been considered. The perpendicularly applied electric field causes a decrease of the azimuthal director angle at the substrate with the conical surface anchoring. In the cells with *d* = 22 *μ*m, the defect loops having the under-twisted and over-twisted areas are formed. At the defect loop the pair of point peculiarities is observed where the 180° jump of azimuthal angle of the director occurs. Under the action of electric field the loops shrink and disappear. In the cells with *d* = 13 *μ*m, the over-twisted and under-twisted defect lines are formed. Applied voltage results in the shortening of lines or/and their transformation into a defect of the third type. The director field distribution near defect lines of three types has been investigated by the polarising microscopy techniques. It has been revealed that the length ratio between the over-twisted and third-type defect lines can be controlled by the electric field.

## Introduction

Liquid crystals (LCs) possess the orientational order that determines an anisotropy of their dielectric, optical, elastic and other properties^1^. Orientational LC structure is quite sensitive to the effect of an external electric or magnetic field^2^, variation of temperature^3^, change of composition and structure of interface^4,5^ and insertion of micro- or nano-objects into liquid crystals^6^. On the other hand, the reverse effect of the formed structure on the ordering or movement of these particles owing to elastic interaction is also characteristic of LC systems (active soft matter)^7–10^. Cholesteric liquid crystals (CLCs) have unique orientational and structural properties that causes a formation of the various director configurations, as well as their transformation and switching^11^. CLC helicoidal structure in which the director (the unit vector **n** oriented along the preferred orientation of the long axes of LC molecules) turns by 2*π* at the distance of helix pitch *p* is formed in a free state. Cholesterics are of great interest for practical applications as controllable diffraction gratings^12–17^, tunable lasers^18,19^, chemical sensor^20^, templates for nanoparticles arrangements with tunable length scales^21^, etc. The formed structure depends mainly on the material parameters of cholesteric, the ratio between CLC layer thickness *d* and helix pitch *p*, the boundary conditions^3,11,22^. Nowadays, the structures forming in the CLC cells with tangential (planar)^3,23–25^, homeotropic^26–28^, homeoplanar^29–33^ boundary conditions and in the cells with surface topography^34–36^ are well studied.

**Figure 1:**
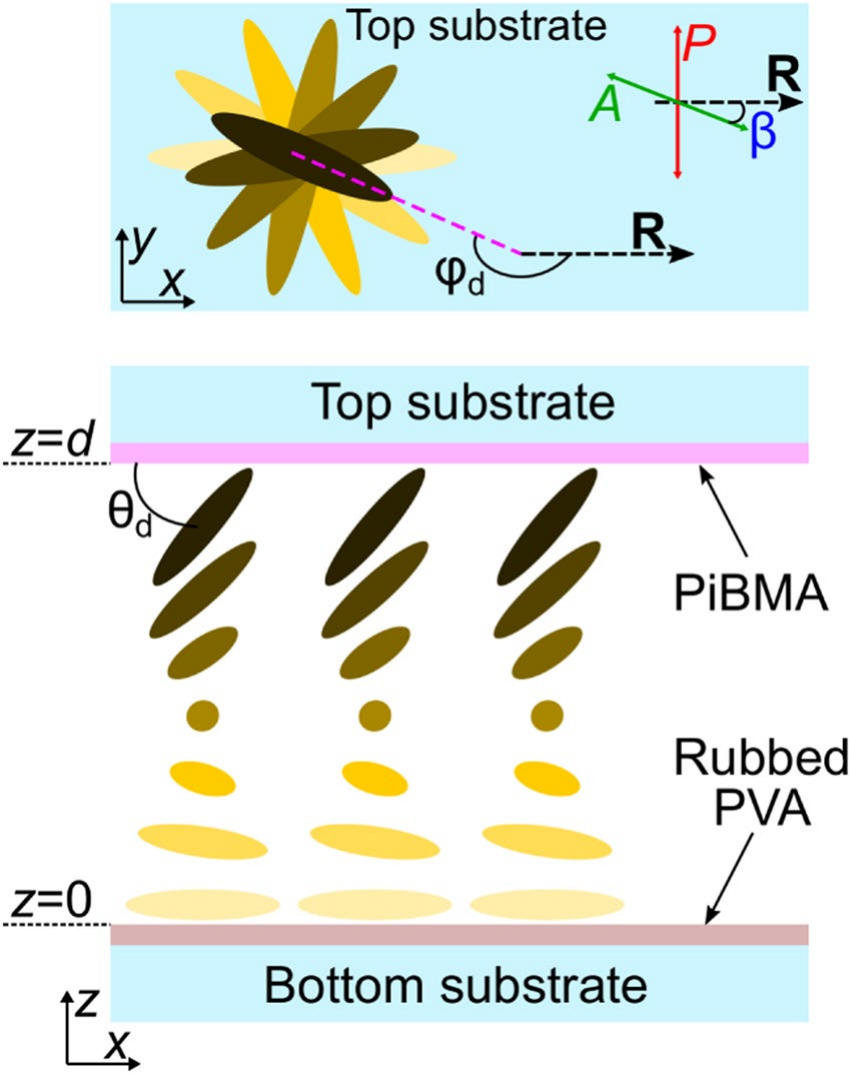
Schemes of the experimental cell and the director orientation in it. Uniform tangential boundary conditions are at the bottom substrate and conical surface anchoring is at the top one. *φ*_*d*_ and *θ*_*d*_ are azimuthal and polar director angles at the top substrate, respectively. The *β* is angle between analyser *A* and rubbing direction **R**.

The cholesteric structures with conical or tangential-conical boundary conditions (uniform tangential boundary conditions at one substrate and conical anchoring at another one) are less studied. So, under the planar surface anchoring at one of the substrates and weak conical anchoring at another one, assigned by own isotropic phase of CLC (the wetting transition), the almost uniform structure or periodic structure of lines (stripes) is formed in case of relatively thin or thicker wetting layer, respectively^37^. Recently, we have studied the cholesteric structures with tangential-conical boundary conditions (Fig. 1), in which the conical anchoring at one of the substrates was specified by the polymer film^38^. In this case, the twisted defect-free structure or the structure with defect walls is formed in the cell at the values of *d*/*p* less than 0.28 (*d* ≅ 6.5 *μ*m). At *d*∕*p* ≥ 0.60 the periodic structure with the period approximately equal to the double helix pitch is formed. The twisted structure with the nonsingular (virtual) surface defects looked like loop or lines whose ends are located at the lateral edges of the CLC layer is realised at the intermediate values of 0.28 < *d*/*p* < 0.6. The effect of electric field on such cholesteric structures has not been previously studied. In the present paper, we have considered the transformations of the defects in the twisted cholesteric structure with tangential-conical boundary conditions under the electric field.

## Results and Discussion

### Defect loop structure

**Figure 2:**
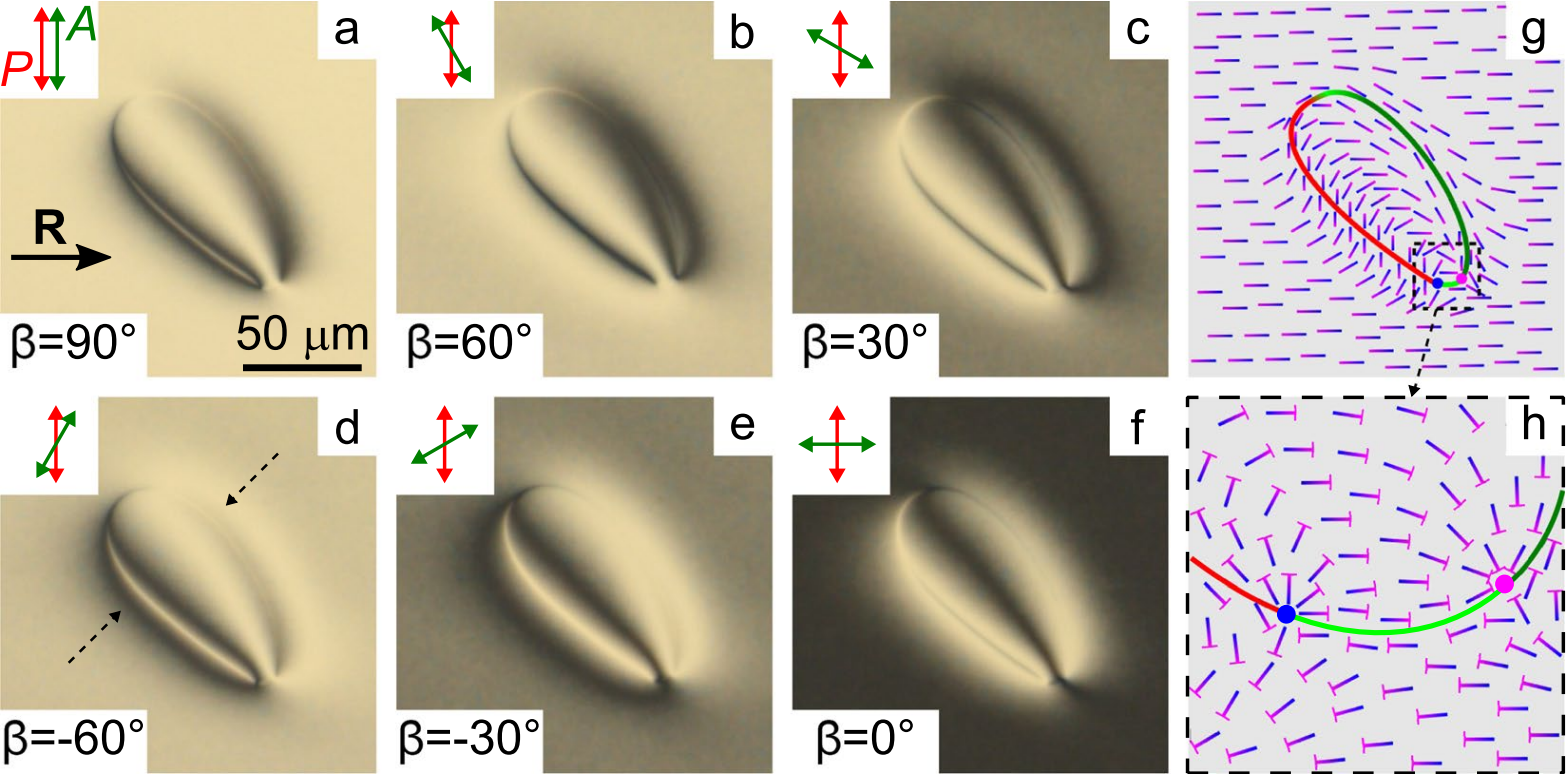
POM photos of the defect loop taken for the angles *β* = 90° (**a**), 60° (**b**), 30° (**c**),  −60° (**d**),  −30° (**e**), 0° (**f**) between the rubbing direction **R** of bottom substrate and analyser *A*. Scheme of the director orientation at the top substrate with conical anchoring (**g**) and near pair peculiarities (blue and pink circles) (**h**). The red and dark green lines indicate the parts of the virtual defect line with under-twisted and over-twisted CLC, respectively. The green line is the part of defect line where the azimuthal twist angle of cholesteric is close to the equilibrium value. Polariser *P* is orthogonal to the rubbing direction, the thickness of the CLC layer *d* is 22 *μ*m. Hereinafter, the ratio *d*/*p* is 0.57, the polariser’s directions are noted by the double arrows, the single arrow indicates the rubbing direction **R**.

The cholesteric under tangential-conical boundary conditions in certain range of *d*/*p* has a twisted structure with the virtual defects which look like the elongated loops or the pairs of lines whose ends are located at the lateral edges of the LC layer when the sample is observed using a polarising microscope^38^. The optical textures of CLC layer with *d* = 22 *μ*m and *d*/*p* = 0.57 are presented in Fig. 2. The rotating analyser method is convenient to analyse the director azimuthal orientation at the substrate with conical surface anchoring (Fig. 2a–f). If the Mauguin condition^39^ is valid, then the incident light linearly polarised perpendicular to the director at the input substrate after passing through twisted orientational structure remains practically linearly polarised with the polarisation perpendicular to the director at the output substrate. In turn, the polarisation direction of transmitted light can be determined by the analyser rotation. The dark areas of optical texture (extinction bands) show where the polarisation of transmitted light perpendicular to the analyser and, consequently, the azimuthal orientation of director at the output substrate can be found (Fig. 1). The Mauguin condition is valid at *p*Δ*n*_eff_/*λ* > > 1, where Δ*n*_eff_ = 0.191 is the effective value of birefringence of LC taking into account the director tilt angle. For sample with *d* = 22 *μ*m the Mauguin number is equal to 10.6 at *λ* = 0.7 *μ*m, therefore the rotating analyser method is suitable for this cell (Fig. 2). It should be noted that the rotating analyser method is suitable to determine the director azimuthal orientation even though the Mauguin condition is slightly broken (more details see in section Methods and Supplementary Figs. S1 and S2). For LC cell presented in Fig. 2 the input substrate was the substrate with tangential anchoring and linear polarisation of light was perpendicular to the rubbing direction **R**. The dark area is observed far from the defect loop (Fig. 2f) when the analyser *A* is approximately parallel to the rubbing direction **R**. It means that the equilibrium azimuthal twist angle *φ*_*d*_ of director at the top substrate with conical surface anchoring is about 180°. The conducted analysis by the rotating analyser method (Fig. 2a–f) revealed that near the defect loop, the azimuthal twist angle *φ*_*d*_ differs from the equilibrium value of 180°. The obtained azimuthal orientation of director is presented in Fig. 2g,h. Since the CLC under study is left-handed then in the left area near the defect loop the CLC is under-twisted (*φ*_*d*_ < 180°) while in the right area near the defect loop the CLC is over-twisted (*φ*_*d*_ > 180°) in comparison with the azimuthal twist angle of CLC far from defect. Additionally, at the substrate with conical surface anchoring, the director at the loop border is parallel to the line of defect.

**Figure 3:**
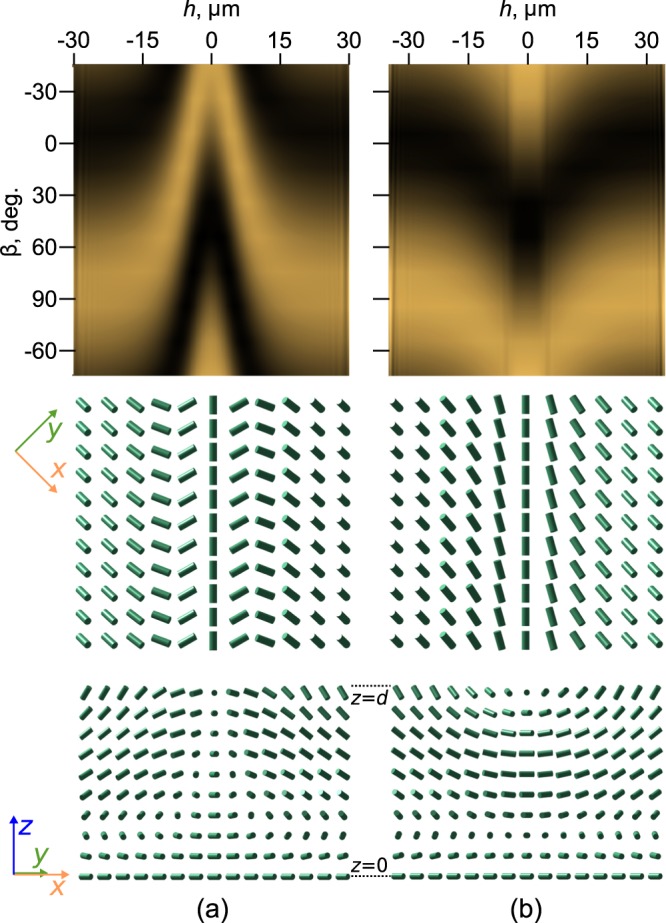
Schemes of the director orientation (middle and bottom rows) and calculated dependences of the extinction bands position observed in polarised white light on the *β* angle (top row) for under-twisted (**a**) and over-twisted (**b**) areas of cholesteric near the defect loop. The middle and bottom rows present the director configuration at the substrate with conical surface anchoring and in the section perpendicular to the cell substrates, respectively. The rubbing direction **R** is parallel to the *x*-axis . Defect lines are oriented at 45° to the rubbing direction **R**, *h* is a distance from the centre of the virtual defect line.

The schemes of director orientation for under-twisted and over-twisted areas near the defect loop are presented in Fig. 3. The dashed arrows in Fig. 2d indicate these regions. The director orientation far from defect loop was calculated by free energy minimisation procedure. Near the defect loop the values of the polar and azimuthal angles of the director were analytically extrapolated based on the experimentally measured values of *φ*_*d*_(*h*), where *h* is a distance from the centre of the defect line^38^. For obtained orientational structures the dependences of the extinction bands position observed in polarised white light on the *β* angle (angle between rubbing direction **R** and analyser) were calculated (Fig. 3(top row)). The calculated dependences are in a good agreement with the transformation of extinction bands observed at the rotation of analyser (Fig. 2a–f).

The defect loop has two closely located peculiarities (the bottom right part of the loop in Fig. 2) from which a pair of extinction bands appears in the certain range of *β* angles. The extinction bands near the under-twisted area of cholesteric appear from one point, while the extinction bands near the over-twisted area appear from another point (Fig. 2a–f). Figure 2h demonstrates the scheme of director orientation near these points obtained by the rotating analyser method. One can see that the tilt angle of the director *θ*_*d*_ has different signs in the vicinity of two points. The director projection near the points at the inner side of the loop has a radial structure and corresponds to the half of radial or hyperbolic boojums with strength  + 1 while outside the loop the director projection corresponds to the half of boojum with strength  − 1. When moving along the border of defect loop the 180° jump of azimuthal angle of director occurs in each point peculiarities and its total change is equal to zero after the moving along the whole loop. Thus, the homogeneous tangential orientation of the director at the bottom substrate is matched with an azimuthal change of the director orientation near the loop defect at the top substrate.

### Defect transformation under electric field

**Figure 4:**
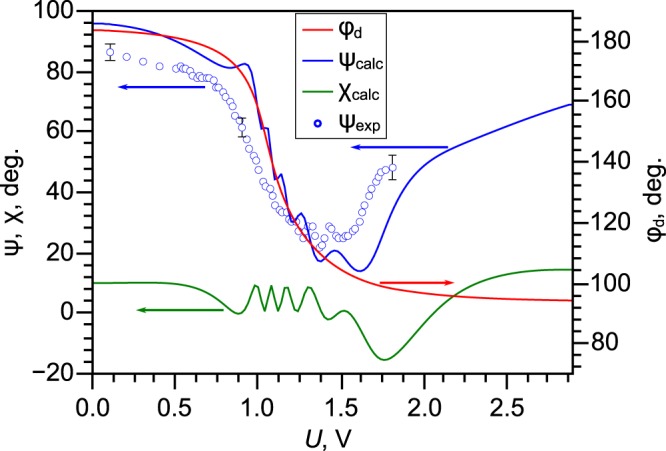
The calculated dependences of *φ*_*d*_ azimuthal director angle at the substrate with conical anchoring (red line), the azimuth of polarisation *ψ*_*calc*_ (blue line), and the ellipticity angle *χ*_*calc*_ (dark green line) on the value of applied voltage *U*. The measured voltage-dependence of polarisation azimuth *ψ*_*exp*_ (blue circles). The calculations and measurements were carried out for the cell with *d* = 22 *μ*m under illumination by green light (*λ* = 546 nm).

The cholesteric liquid crystal under study has a positive dielectric anisotropy Δ*ε*. In this case, the electric field applied perpendicular to the substrates causes a change of both the polar and azimuthal angles of the director, including the azimuthal twist angle *φ*_*d*_ of director at the substrate with conical surface anchoring. For the twisted structure without defects, the calculated dependences of *φ*_*d*_ angle and polarisation characteristics of transmitted monochromatic light with wavelength *λ* = 546 nm on the value of applied voltage *U* are shown in Fig. 4. One can see that the applied electric field induces the change of *φ*_*d*_ and the most significant variation is observed in the range of voltages from 0.5 to 1.5 V where *φ*_*d*_ angle decreases from 183° to 94°. The change of polar angle of the director in the bulk leads to decrease of CLC layer birefringence and the breaking of the Mauguin regime. In addition, the transformation of director configuration results in the nonmonotonic dependence of the polarisation azimuth *ψ*_*calc*_ of transmitted light on the applied voltage. Oscillations of the ellipticity angle *χ*_*calc*_ and azimuth of polarization *ψ*_*calc*_ result from decreasing the effective CLC birefringence. These oscillations are described by the equations (19), (20) and (26) in ref. ^40^ and demonstrated in Fig. 4.6 in ref. ^39^. The experimentally measured dependence *ψ*_*exp*_(*U*) for transmitted monochromatic light with wavelength *λ* = 546 nm shows similar nonmonotonic behaviour. At that, a noticeable deviation from the Mauguin regime was observed for *U* ≥ 1.4 V which is in a good agreement with the simulation data.

Due to the above-mentioned variations of the polar and azimuthal angles of director induced by the action of electric field, the defect loops and defect lines become unstable and change. Two scenarios of transformations can be observed: *(i)* a change of loop size or line length and *(ii)* a change of defect line structure accompanied by the formation of new type of defect.

#### Change of defect size

The change of the loop size results from the counter motion of the loop parts with an azimuthal twist angle of CLC close to the equilibrium value (green lines in Fig. 2g) and, for this reason, the shape of the defect loop becomes less elongated (see Supplementary Fig. S3). If the electric field is switched off during the transformation process then the size and form of the defect loop slightly change. In addition, the part of loop with a pair of peculiarities becomes sharper. The application of voltage to the LC cell during a sufficiently long time (tens of minutes) leads to the disappearing of the defect loop and the defect-free structure is retained for several days after switching off the electric field.

**Figure 5:**
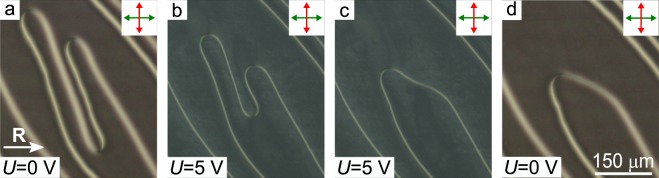
POM photos of CLC layer with defect lines in the initial state (**a**), in 25 s (**b**), in 60 s (**c**) after application of voltage *U* = 5 V and after switching off the electric field (**d**). The thickness of LC layer is 13 *μ*m.

Besides the defect loops, the pairs of defect lines are observed in the samples under study. At that, the number of defect lines increases when the thickness of the CLC layer decreases. Near the pair of defect lines, the director orientation changes in the same way as for the case of the defect loop, i.e. over-twisted and under-twisted areas of CLC are observed. Generally, the lines turn into each other by the formation of bends, in which the azimuthal twist angle of CLC is close to the equilibrium value far from defects. The application of the electric field initiates the movement of these bends similarly to the motion of the loop areas where the azimuthal twist angle of CLC is close to equilibrium value. It causes the straightening and decreasing the length of defect lines (Fig. 5b–d). The application of voltage to the LC cell during a sufficiently long time (tens of minutes) leads to the disappearing the almost all defect lines and the defect-free structure is retained for a long time (several days) without applied voltage.

#### Change of defect type

**Figure 6:**
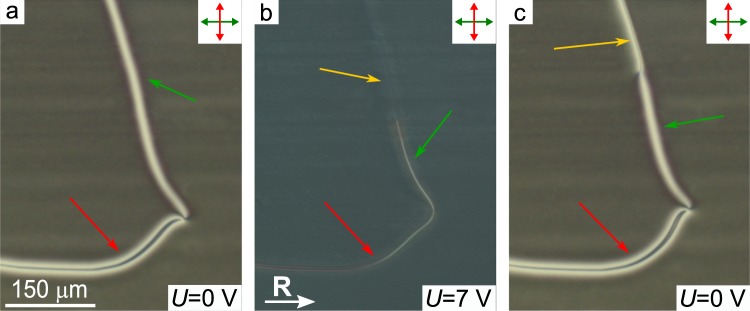
POM photos of CLC layer with defect lines in the initial state (**a**), under application of voltage *U* = 7.0 V (**b**) and after switching off the electric field (**c**). The thickness of CLC layer is 13 *μ*m. The over-twisted, under-twisted and third-type defect lines are indicated by green, red and yellow single arrows, respectively.

**Figure 7:**
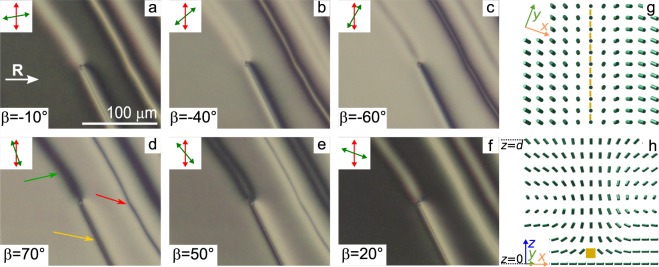
POM photos of CLC layer with three types of the defect lines taken after switching off the voltage *U* = 7.0 V. Angle *β* is −10° (**a**), −40° (**b**), −60° (**c**), 70° (**d**), 50° (**e**), 20° (**f**). Scheme of the director orientation near the third-type defect line at the top substrate with conical surface anchoring (**g**) and the director configuration in the section perpendicular to the cell substrates and defect line (**h**). The thickness of CLC layer is 13 *μ*m. The over-twisted, under-twisted and third-type defect lines are indicated by green, red and yellow single arrows, respectively.

Applied voltage induces the decreasing of the azimuthal angle of director far from defects both in the CLC bulk and at the substrate with conical boundary conditions (Fig. 4). It, in turn, influences the director’s distribution near the defects but does not change the orientation of defect lines (Fig. 5). The difference between azimuthal angles of director far from defect and near the under-twisted defect line decreases while for the over-twisted defect line the similar difference increases. Under sufficiently high voltage (about 7 V) the area with significant deformation of director is formed near the over-twisted defect line, that leads to the appearing of the third-type defect line (Fig. 6). Generally, the transformation begins in a single point and then the length of the third-type defect line increases along the over-twisted defect. After the total change of the over-twisted defect line the transformation of the under-twisted defect line occurs. The rate of transformation process rises when the value of applied voltage increases. When the value of applied voltage decreases the third-type defect line remains stable up to *U* = 1.9 V. However, further decreasing of voltage to 1.8 V leads to the fast (during a few seconds) inverse transformation of the under-twisted defect line. The inverse transformation of the over-twisted defect line occurs at voltages less than 1.6 V and this process takes tens of seconds. If the voltage *U* ≅ 1.6 V is applied before the relaxation process end then the transformation is stopped. In this case, only the over-twisted defect line is transformed into the third-type defect line in the range from 1.7 to 2.4 V. At the applied voltages *U* > 2.4 V the change of under-twisted defect line is observed too. The observed hysteresis of the defects transformation makes it possible to control the position of the point dividing the over-twisted and the third-type defect lines by increasing/decreasing of voltage and fix this point using the value of voltage about 1.6 V (see Supplementary Movie 1). Since the transformation between over-twisted and third-type defect lines is a slow process the azimuthal angle of director near the third-type defect can be determined by the rotating analyser method in the case when the electric field is switched off (Fig. 7).

Figure 7 demonstrates the cell area with all three types of defect lines after switching off the voltage. The POM photos were taken for the various *β* angles and it allows determining the azimuthal distribution of the director at the top substrate with conical surface anchoring. As in the case of the defect loop, the pairs of extinction bands near the under-twisted and over-twisted defect lines is observed. For the under-twisted defect line the pair of extinction bands can be seen for negative values of *β* angles (Fig. 7b), whereas for the over-twisted defect line this pair appears for positive *β* angles (Fig. 7e). The pair of extinction bands is symmetrically placed with respect to each defect line. It means the identical azimuthal director orientation from the left and right of the defect line^38^.

**Figure 8:**
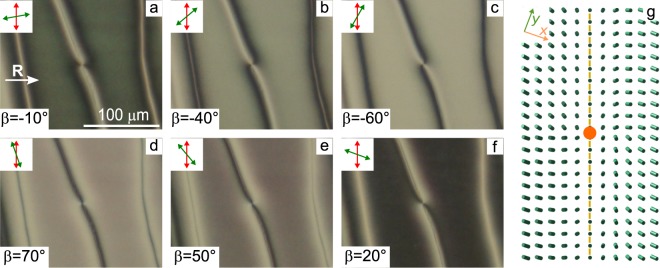
POM photos of the third-type defect line with the reversing point. Angle *β* is −10° (**a**), −40° (**b**), −60° (**c**), 70° (**d**), 50° (**e**), 20° (**f**). The thickness of CLC layer is 13 *μ*m, the ratio *d*/*p* is 0.57. Scheme of the director orientation near the third-type defect line with reversing point (orange circle) at the substrate with conical anchoring (**g**).

A different pattern is observed for the third-type defect where only one extinction band is seen near the line (Fig. 7). For negative *β* angles the extinction band is placed to the right of defect line (Fig. 7a–c), while for positive *β* angles the extinction band is located to the left of defect line (Fig. 7d–f). It means that the transformation of over-twisted defect line under the action of electric field is accompanied by 180° azimuthal rotation of director from one side of the defect line (in Fig. 7 the right side of the defect line). This azimuthal rotation of the director can be explained by the change of a polar angle of the director at the substrate with conical anchoring near the defect line. It leads to the appearance of the linear defect near the substrate with tangential anchoring (Figs. 7 and 8). In the experiment this defect is clearly seen both in the crossed polarisers and the single polariser geometry for any orientation of the polariser relative to the rubbing direction (see Supplementary Fig. S4). On the contrary, in the single polariser geometry the under-twisted and over-twisted defect lines are seen when the polariser is oriented parallel to the rubbing direction **R** and are almost invisible when the polariser is orthogonal to **R**. In the single polariser geometry, the change of the optical patterns of the defect lines under the action of the electric field is also different. So, the under-twisted and over-twisted defect lines become sharper and they are seen even when the polariser is oriented perpendicularly to the rubbing direction (see Supplementary Fig. S4). It is explained by the increasing the refractive index gradient because the polar and azimuthal angle of director far from defect lines change, but the director orientation at the defect lines remains unchanged. On the contrary, the third-type defect line is almost invisible for any polariser orientation owing to the decreasing the gradient of refractive index (see Supplementary Fig. S4). The scheme of director orientation near the third-type defect line based on the analysis of optical patterns is shown in Fig. 7g,h.

The above-described azimuthal rotation of the director under the action of electric field is possible to the left or to the right of defect line. In the experiment, both variants of the third-type defect can be realised even for the same line. In this case, the reversing point is formed at the defect line (Fig. 8).

## Conclusion

Influence of an electric field on the CLC structure and defects formed in the cholesteric layer with tangential-conical boundary conditions has been considered. The samples with confinement ratio *d*/*p* = 0.57 and two thicknesses (13  *μ*m and 22  *μ*m) of CLC layer have been investigated. The twisted structure with elongated defect loops is formed in the thicker CLC layer. The equilibrium azimuthal twist angle of the director *φ*_*d*_ = 180° at the top substrate with conical surface anchoring has been determined by the rotating analyser method. The defect loop has the under-twisted (*φ*_*d*_ < 180°) and over-twisted (*φ*_*d*_ > 180°) areas. At the defect loop the pair of point peculiarities is observed where the 180° jump of azimuthal angle of the director occurs. Thus, the total change of the azimuthal angle is equal to zero after moving along the whole loop. It allows matching a various azimuthal orientation of the director near the defect loop at the substrate with the conical surface anchoring and homogeneous director orientation at the substrate with tangential boundary conditions. Under the action of electric field the angle *φ*_*d*_ far from defect decreases and it leads to the instability of the over-twisted area. As a result, the loop size decreases and the defect finally disappears. If the electric field is switched off before its disappearance, then the actual loop size is memorised. So, the application of the electric field allows to eliminate all defects and obtain a defect-free twisted structure which is stable for a long time.

The over-twisted and under-twisted defect lines are formed in the sample with thinner CLC layer. The applied electric field causes two kinds of the defects changes: *i*) a decrease of the defect lines length; *ii*) a change of defect line structure accompanied by the formation of the third-type defect. The length of the lines decreases up to the total disappearance and defect-free structure is retained for a long time after switching off the electric field. The third-type defect line formed under the action of the electric field is characterised by the preferably perpendicular orientation of the director to the substrate at the defect line and 180° change of the azimuthal angle *φ*_*d*_ in the adjacent areas. After switching off the electric field, the inverse transformation of the over-twisted defect line occurs for tens of seconds. The application of voltage before the end of the relaxation allows controlling the length ratio between the over-twisted and third-type defect lines. The obtained results can be interesting for the development of LC systems with controllable defects and the electrically-operated interface.

## Methods

The experiment was carried out with sandwich-like cells consisting of two glass substrates with transparent ITO electrodes coated with polymer films. The bottom substrate of cell was covered by the polyvinyl alcohol (PVA) (Sigma Aldrich) and the top substrate was covered by the poly(isobutyl methacrylate) (PiBMA) (Sigma Aldrich) (1). The polymer films were deposited on the substrates by spin coating. The PVA film was unidirectionally rubbed while the PiBMA film was without additional treatment after the deposition process. The LC layer thicknesses *d* = 13  *μ*m or *d* = 22  *μ*m assigned by the glass microspheres (Duke Scientific) were measured by means of the interference method with spectrometer HR4000 (Ocean Optics) before the filling process. The nematic mixture LN-396 (Belarusian State Technological University) doped with the left-handed chiral additive cholesteryl acetate (Sigma Aldrich) was used as a cholesteric. The cells were filled by the CLC in the mesophase at room temperature. After the filling process, the cells were kept for at least 24 h before measurements. In the CLC cells under study the tangential-conical boundary conditions with the *θ*_*d*_ tilt angle at the substrate with PibMA close to 50° are formed^41,42^. The cholesteryl acetate contents were chosen to obtain the confinement ratio *d*/*p* = 0.57 for both thicknesses of LC layer. The samples were studied by means of the polarising optical microscope (POM) Axio Imager.A1m (Carl Zeiss) using white light or quasi-monochromatic light with wavelength *λ* = 546 nm produced by the interference filter. An alternating voltage of 1 kHz frequency and variable amplitude was applied to the cells using the signal generator AHP-3122 (Aktakom).

The director orientation in LC layer was calculated by means of the free energy minimisation^43^. The Frank elastic energy density *F*_*k*_ was expressed as:

1 $${F}_{k}=\frac{1}{2}{k}_{11}{(\nabla \cdot {\bf{n}})}^{2}+\frac{1}{2}{k}_{22}{({\bf{n}}\cdot \nabla \times {\bf{n}}+2\pi /p)}^{2}+\frac{1}{2}{k}_{33}{({\bf{n}}\times \nabla \times {\bf{n}})}^{2},$$

here **n** is the director, *k*_11_, *k*_22_ and *k*_33_ are the splay, twist and bend elasticity coefficients, respectively. In simulation the LC layer was divided into 32 homogeneous sublayers. The following elasticity coefficients were taken: *k*_11_ = 11.1 pN, *k*_22_ = 7.6 pN and *k*_33_ = 17.1 pN^38^. The elastic energy of the layer was minimised in the gradient direction. The pair of *θ*, *φ* angles was obtained by the five gradient components. Numerical convergence of this function was controlled by artificial viscosity. The defects of the structures were analytically interpolated between the neighbouring domain structures. The values of *φ*(*x*) were adjusted by ${\varphi }_{0}{\cosh }^{-1}(4x/L)$, where *L* is a characteristic defect thickness and *φ*_0_ were taken from experimental data for each defect. In Fig. 3a  *L* = 22  *μ*m and in Fig. 3b *L* = 17.6  *μ*m.

The effective value of birefringence of LC taking into account the director tilt angle calculated as^40,44^:

2 $$\Delta {n}_{e\mathrm{ff}}=\frac{{n}_{\parallel }}{\sqrt{1+\nu si{n}^{2}{\bar{\theta }}_{s}}}-{n}_{\perp },$$

where $\nu ={\left(\frac{{n}_{\parallel }}{{n}_{\perp }}\right)}^{2}-1$, and $\bar{{\theta }_{s}}=(1/d){\int }_{0}^{d}\theta (z)dz$ is tilt angle averaged over the thickness of the LC layer^45^ equal to 17. 2°. For *d* = 22 *μ*m the Mauguin number *p*Δ*n*_eff_/*λ* ≅ 10.6 (at *λ* = 0.7 *μ*m). For *d* = 13 *μ*m the Mauguin number is equal to 6.2 at *λ* = 0.7 *μ*m, at that for short-wave region the Mauguin condition is valid (*p*Δ*n*_eff_/*λ*≅ 10.7 at *λ* = 0.4 *μ*m), i.e. the Mauguin condition is broken slightly.

The optical properties of LC structures were calculated by Finite-Difference Time-Domain (FDTD) method implementation in commercial Lumerical package. The LN-396 uniaxial crystal was characterised by *n*_∥_ = 1.741 and *n*_⊥_ = 1.528. LC structure was illuminated from below by the plane wave with normal incidence along the *z*-axis and linear polarisation perpendicular to the rubbing direction. Periodic boundary conditions were applied at the lateral boundaries of the simulation box (along the *x* and *y* axes), while the perfectly matched layers (PML) were used on the remaining top and bottom sides. POM images were obtained for the white light with a spectral distribution similar to the spectrum of the light source.

## Supplementary information

Supplementary Movie.

Supplementary Information.
