# Supplementary material for: Electrically induced transformations of defects in cholesteric layer with tangential-conical boundary conditions
Source: Sci Rep. 2020 Mar 17;10:4907. doi: 10.1038/s41598-020-61713-9 (PMC7078221; doi:10.1038/s41598-020-61713-9)

Supplementary Information

Electrically induced transformations of defects in cholesteric layer with tangential-conical boundary conditions

Mikhail N. Krakhalev*[1,2], Oxana O. Prishchepa[1,2], Vitaly S. Sutormin[1,2]
Rashid G. Bikbaev[1,2], Ivan V. Timofeev[1,2], and Victor Ya. Zyryanov[1]

[1] *Kirensky Institute of Physics, Federal Research Center KSC SB RAS, Krasnoyarsk 660036, Russia*

[2] *Institute of Engineering Physics and Radio Electronics, Siberian Federal University, Krasnoyarsk 660041, Russia*

Corresponding author: M.N. Krakhalev, e-mail: kmn@iph.krasn.ru

Supplementary Figures 1-4

Supplementary Figure 1| POM photos of CLC layer with virtual defect lines taken for the angles β = 90° (a), 60° (b), 30° (c), -60° (d), -30° (e), 0° (f) between the rubbing direction **R** of bottom substrate and analyser *A*. Polariser *P* is orthogonal to the rubbing direction. The thickness of the CLC layer *d* is 13 μm and the ratio *d*/*p* is 0.57. The polariser's directions are noted by the double arrows, the single arrow indicates the rubbing direction **R**. POM photos taken in white, green (λ = 546 nm), orange (λ = 602 nm), and dark-red (λ = 673 nm) light. The positions of the extinction bands observed under different wavelengths and white light coincide with an accuracy of 0.5 μm.

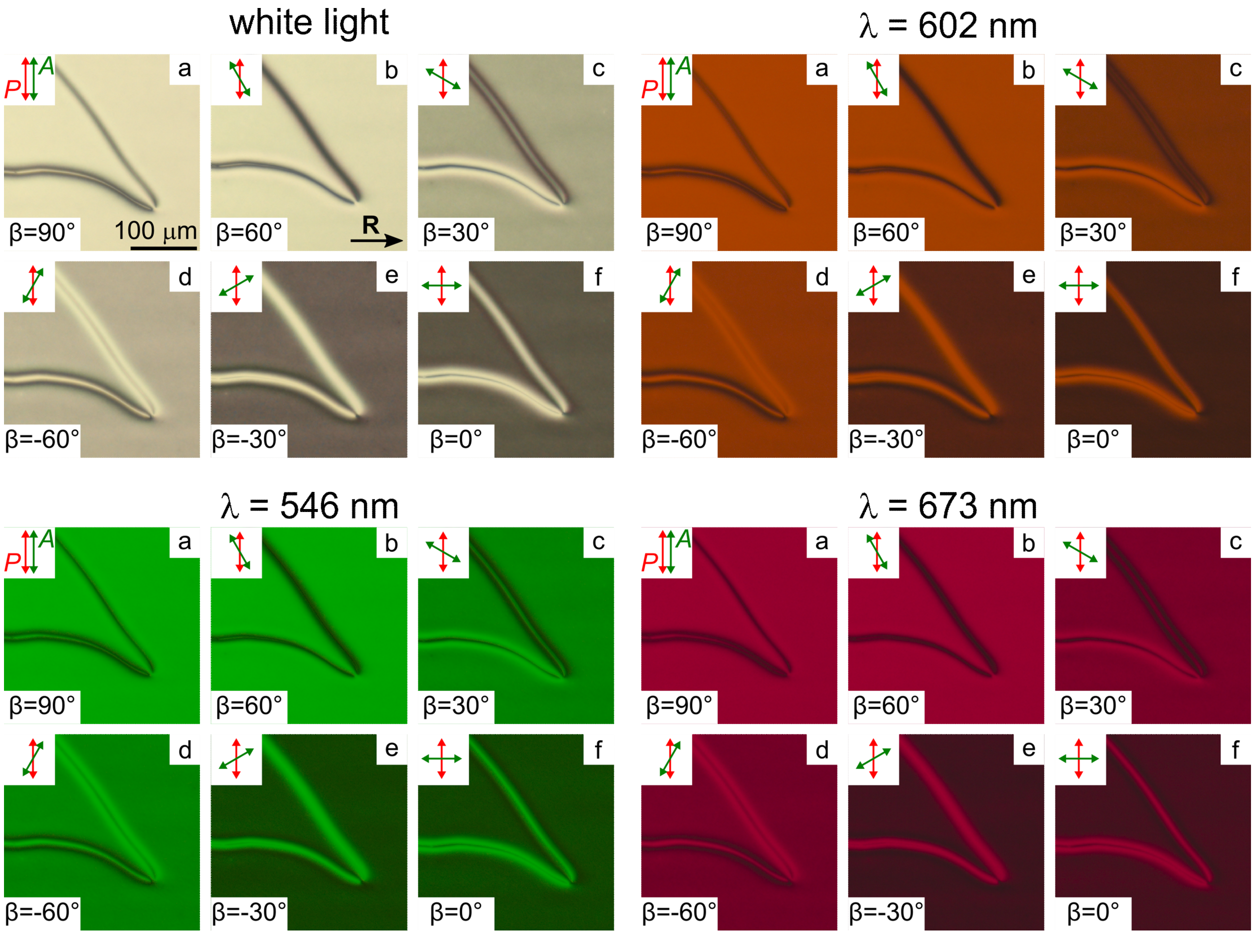

Supplementary Figure 2| Light intensity profiles *I*(*h*) measured near the under-twisted defect line for the β = -60° (a) and over-twisted defect line for the β = 30° (b) when the cell is illuminated with white, green (λ = 546 nm), orange (λ = 602 nm), and dark-red (λ = 673 nm) light.

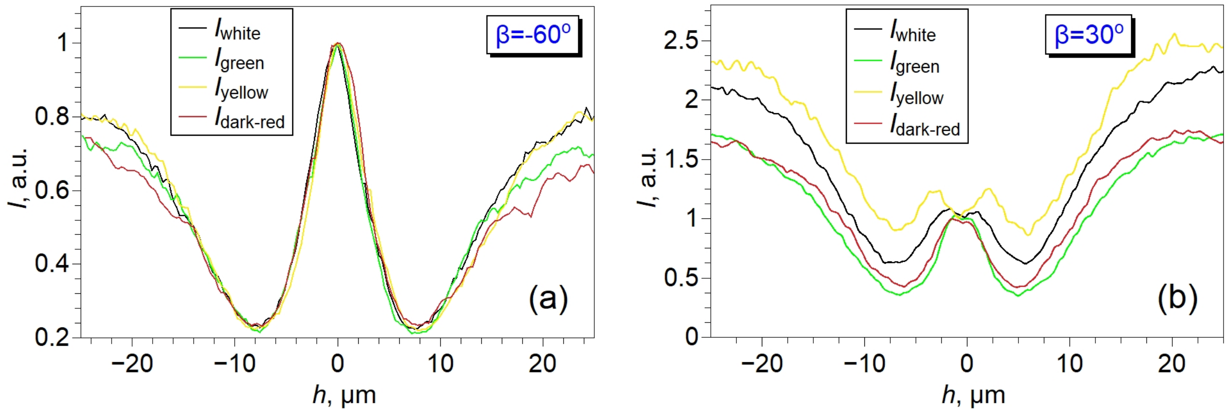

Supplementary Figure 3| POM photos of CLC layer with defect loop in the initial state (a), in 2 min (b), in 6 min (c) after application of voltage $U = 7$ V and after switching off the electric field (d). The optical textures of cholesteric in 30 s after the second application of $U = 7$ V (e) and with final defect-free structure (f). The thickness of CLC layer is 22 μm.

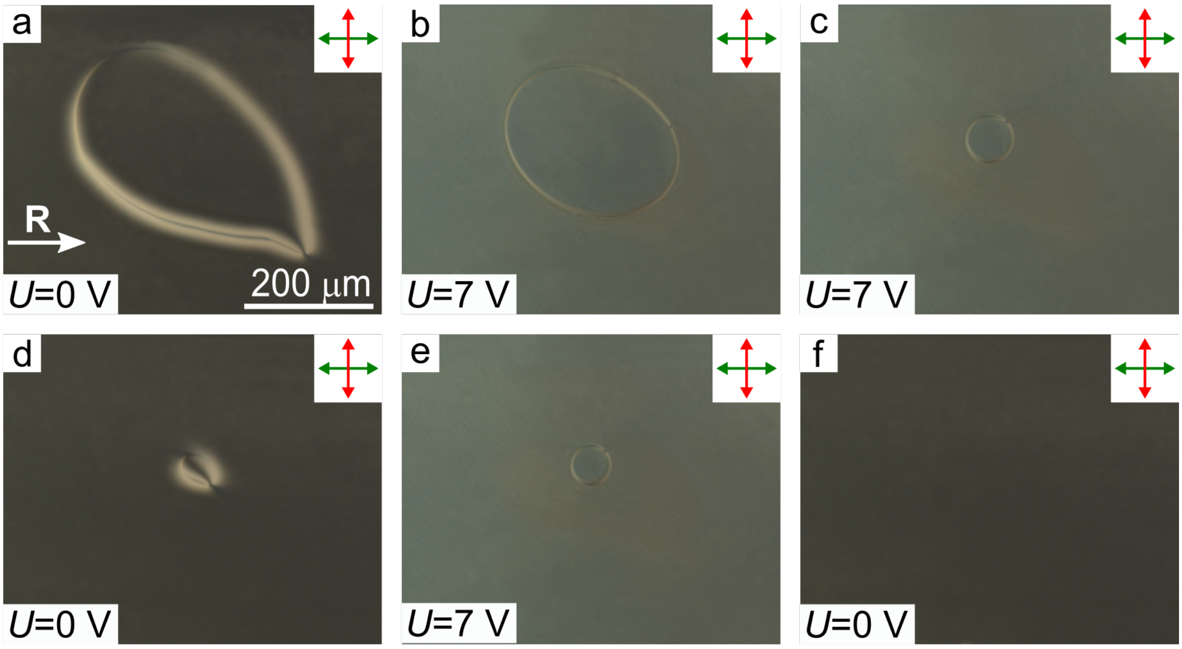

Supplementary Figure 4| POM photos of the area with defect lines taken in the single polariser geometry without the electric field (a), (b) and under application of voltage $U = 1.7$ V (c), (d). The polariser is parallel (a), (c) and orthogonal (b), (d) to the rubbing direction **R**. POM photo of this area taken in crossed polarises under voltage $U = 1.7$ V (e). The thickness of the CLC layer d is 13 μm, the ratio d/p is 0.57, scale bar is 50 μm. The polariser's directions are noted by the double arrows, the black single arrow indicates the rubbing direction **R**. The over-twisted, under-twisted and third-type defect lines are indicated by green, red and blue single arrows, respectively.

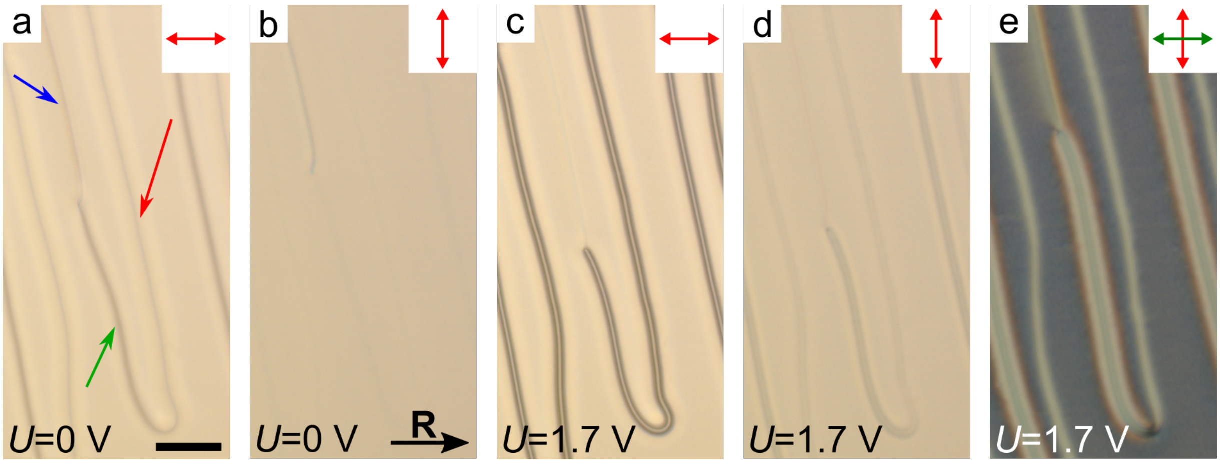

Supplement: Supplementary file 2 — Supplementary Information. [file 41598_2020_61713_MOESM2_ESM.pdf]
